# The Effect of Covid-19 on Alcohol Use Disorder and the Role of Universal Alcohol Screening in an Inpatient Setting: A Retrospective Cohort Control Study

**DOI:** 10.1093/alcalc/agab059

**Published:** 2021-08-21

**Authors:** Mohsan Subhani, Abhishek Sheth, Stuart Unitt, Guruprasad P Aithal, Stephen D Ryder, Joanne R Morling

**Affiliations:** Nottingham Digestive Diseases Biomedical Research Centre (NDDC), School of Medicine, University of Nottingham, Derby Rd, Lenton, Nottingham NG7 2UH, UK; NIHR Nottingham Biomedical Research Centre, Nottingham University Hospitals NHS Trust and the University of Nottingham, E Floor, West Block, Derby Rd, Lenton, Nottingham NG7 2UH, UK; Nottingham Digestive Diseases Biomedical Research Centre (NDDC), School of Medicine, University of Nottingham, Derby Rd, Lenton, Nottingham NG7 2UH, UK; NIHR Nottingham Biomedical Research Centre, Nottingham University Hospitals NHS Trust and the University of Nottingham, E Floor, West Block, Derby Rd, Lenton, Nottingham NG7 2UH, UK; Activity & Access Team, Nottingham University Hospitals NHS Trust, Derby Rd, Lenton, Nottingham NG7 2UH, UK; Nottingham Digestive Diseases Biomedical Research Centre (NDDC), School of Medicine, University of Nottingham, Derby Rd, Lenton, Nottingham NG7 2UH, UK; NIHR Nottingham Biomedical Research Centre, Nottingham University Hospitals NHS Trust and the University of Nottingham, E Floor, West Block, Derby Rd, Lenton, Nottingham NG7 2UH, UK; Nottingham Digestive Diseases Biomedical Research Centre (NDDC), School of Medicine, University of Nottingham, Derby Rd, Lenton, Nottingham NG7 2UH, UK; NIHR Nottingham Biomedical Research Centre, Nottingham University Hospitals NHS Trust and the University of Nottingham, E Floor, West Block, Derby Rd, Lenton, Nottingham NG7 2UH, UK; Nottingham Digestive Diseases Biomedical Research Centre (NDDC), School of Medicine, University of Nottingham, Derby Rd, Lenton, Nottingham NG7 2UH, UK; NIHR Nottingham Biomedical Research Centre, Nottingham University Hospitals NHS Trust and the University of Nottingham, E Floor, West Block, Derby Rd, Lenton, Nottingham NG7 2UH, UK; Division of Epidemiology and Public Health, Nottingham University Hospitals NHS Trust and the University of Nottingham, Hucknall Rd, Nottingham NG5 1PB, UK

## Abstract

**Aim:**

To assess the impact of Covid-19 on alcohol use disorders (AUD) and the role of universal alcohol screening (UAS) in an inpatient setting.

**Methods:**

Retrospective cohorts were defined as pre-pandemic and pandemic admitted to Nottingham University Hospitals (April to October; 2019 and 2020) and had alcohol assessment by AUDIT-C. AUDIT-C score was assessed against age, sex, ethnicity, admission type, speciality and primary diagnosis of mental disorders. Subgroup analysis for Covid-19 positive patients was performed.

**Results:**

A total of 63,927 admissions (47,954 patients) were included. The pandemic period compared to pre-pandemic had fewer overall admissions (27,349 vs 36,578, *P* < 0.001), fewer with AUD (17.6% vs 18.4%, *P* = 0.008) but a higher proportion of alcohol dependents (3.7% vs 3.0%, *P* < 0.0001). In the pandemic those with AUD were more likely to be male (*P* = 0.003), white (*P* < 0.001), in relationship (*P* < 0.001), of higher socioeconomic background (*P* < 0.001), have alcohol-related mental disorders (*P* = 0.002), emergency admission (*P* < 0.001), medical speciality admission (*P* < 0.001) and shorter length of stay (*P* < 0.033) compared to pre-pandemic AUD. Covid-19 positive patients with concomitant AUD died at younger age (*P* < 0.05) than Covid-19 positive patients at low risk for AUD.

**Conclusions:**

The pandemic changed the characteristics of inpatients with AUD. There was a higher proportion of alcohol-dependent admissions with evidence that a younger, less deprived group have been significantly impacted. UAS provides a useful tool to screen for AUD and to identify the change when facing sudden health crises.

## Teaser

During pandemic, there was reduction in total number of acute hospital admissions. Pattern of AUD was similar before and during the pandemic though a higher proportion during the pandemic was alcohol dependent. The AUD screened positive in pandemic were more likely to have mental disorders.

## INTRODUCTION

The Covid-19 pandemic has presented social and healthcare challenges. Public Health Scotland reported a 6% reduction in total volume of pure alcohol sold per adult during the pandemic, but sales in supermarkets and corner shops (‘off-trade sales’) per adult actually increased in Scotland (by 28%) and in England (by 29%) (Public-Health-Scotland, 2021). For the UK as a whole, overall sales of units of alcohol in the first months of 2020 lockdown were not lower than the corresponding periods in 2015–2018 because although pubs and bars were closed purchasing seemed to be transferred to supermarkets (Anderson *et al*., 2021). But there is evidence that home drinking (off-trade sales) is concentrated in population segments and is associated with greater physical and psychological harm. Public Health England estimate that over 8.4 million people were drinking at an increased risk level during this pandemic, compared to 4.8 million before the first national lockdown (Public-Health-England, 2020a).

According to national statistics for England and Wales, in 2020 the death rate due to wholly alcohol attributable conditions reached 12.8 deaths per 100,000, the highest since 2001. During the first three quarters of 2020 (January to September) there were 5460 deaths due to wholly alcohol attributable conditions compared to 4689 deaths for similar period in 2019, a 16.4% increase (ONS, 2021). Probably reflecting the fall in all acute hospital admissions during the early months of Covid, the number of hospital admissions in England related to alcohol fell in 2020 compared to 2019 (NHS-Digital, 2021a). Nevertheless, a tertiary liver unit in London, England, reported that the Covid-19 period was associated with a 2-fold increase (from 19% to 48% *P* = <0.0001) in admissions for alcohol-related liver disease (ARLD) and an increase from 11% to 24% in those cases requiring intensive or high dependency care (Cargill *et al*., 2020).

Although the prevalence of harmful alcohol use is disproportionately high among hospitalized patients, it is persistently underdiagnosed and undertreated (Schneekloth *et al*., 2001). The UK National Confidential Enquiry into Patient Outcome and Death for 2013 highlighted the failure to screen and refer hospitalized patients adequately for alcohol use disorder (AUD) (Juniper *et al*., 2013). Its recommendations included universal screening of people presenting to the hospital using a validated tool such as AUDIT-C.

Although UK data mentioned above show an increase in off-trade sales (i.e. home consumed) alcohol consumption and alcohol-related mortality, we present here a more detailed analysis of the impact of Covid-19 on AUD among hospitalized patient using universal alcohol screening (UAS) data by AUDIT-C score in an inpatient setting.

## METHODS

We analysed retrospectively collected data at Nottingham University NHS hospitals (NUH), England, UK which serve a population of 700,000. Approval was obtained from the local audit department (Registration Number: 20-728C).

**Fig. 1:**
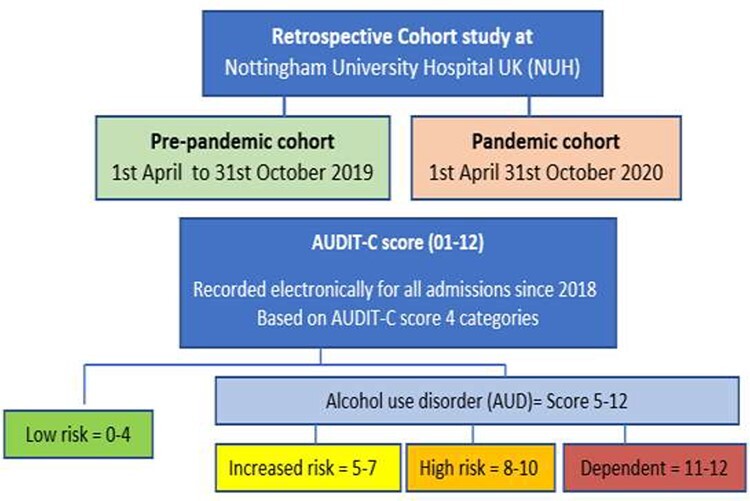
Description of pre-pandemic and pandemic cohorts; AUDIT-C score risk categories.

Two admission cohorts were defined as: ‘pre-pandemic’ (1 April 2019 to 31 October 2019) and ‘pandemic’ (1 April 2020 to 31 October 2020) (Fig. 1). We identified all hospital admissions for the two periods and applied following study eligibility criteria: (a) adult aged 16 years and over, and (b) having had an alcohol assessment using AUDIT-C score.

UAS has been electronically recorded for all admissions to NUH since 2018. All patients admitted to NUH have a mandatory alcohol assessment by AUDIT-C score (which ranges from zero to max. 12) by a qualified nurse within 24 h of admission. We divided patients into four groups: low risk (AUDIT-C score 0–4), increased risk (AUDIT-C score 5–7), high risk (AUDIT-C score 8–10) and alcohol dependent (AUDIT-C score 11–12). An AUDIT-C score of ≥5 was considered a positive screen for AUD (Fig. 1).

The primary aim was to describe the impact of the COVID-19 pandemic on AUD as identified by AUDIT-C score among hospitalized patients, identify shared characteristics and establish UAS acceptance rates among patients. The secondary aim was to compare rates of AUD risk groups between pandemic-cohort and pre-pandemic cohort.

The term ‘AUD’ was used to represent and discuss the results of AUDIT-C score and ‘alcohol disorders (AD)’ to encompass broader alcohol-related problems including ARLD.

### Variables

Data for all hospital admissions during defined study periods were extracted from electronic medical records. A standardized proforma was designed to extract anonymized data on age, gender, ethnicity, civil status, mode of admission, AUDIT-C score at admission, primary diagnosis of mental and behavioural disorder due to alcohol (ICD-10 version 5), inpatient speciality of care (medical vs surgical, Supplementary Table SS3), length of stay (LOS), number of hospital admissions, inpatient mortality and indices of multiple deprivation (IMD). For IMD, the English indices of deprivation 2015 guide were used. The index of multiple deprivation decile (IMDD) combines information from seven domains and produces an overall measure of deprivation. IMD ranks the scores to produce quintiles with 1 equal to most deprived 20% and 5 equal to least deprived 20% neighbourhoods nationally. Civil status was defined as ‘in relationship’ (married, in civil partnership or in long term relationship) and ‘not in relationship’ (single, divorced, separated, dissolved civil partnership, widowed or surviving civil partner).

A subgroup analysis was performed on the pandemic cohort for Covid-19 positive and negative patients, including inpatient mortality analysis between Covid-19 positive AUD vs Covid-19 positive low risk for AUD. In NUH, the diagnosis of Covid-19 is confirmed by accepted molecular tests and/or by radiology.

### Statistical analysis

The normally distributed quantitative variables were summarized as mean ± standard deviation (SD) and the quantitative variables which did not follow a normal distribution as median ± range (minimum and maximum). The categorical variables were summarized as absolute and relative frequencies ±95% confidence interval. The relationship between AUDIT-C score and normally distributed quantitative variables was assessed by parametric tests (Pearson’s correlation coefficient, unpaired *T*-test, ANOVA test) and non-normally distributed by non-parametric tests (Spearman’s correlation coefficient, Mann–Whitney *U* test, Kolmogorov–Smirnov test). Categorical variables were analysed by the Chi-squared test, with results reported as number (percentage).

Statistical analysis was conducted using Statistical Package for the Social Sciences (SPSS version 26.0) and Prisma GraphPad (version 8.0). STROBE reporting guidelines for reporting observational studies in epidemiology were used throughout the article.

## RESULTS

### Description of the cohorts

**Fig. 2:**
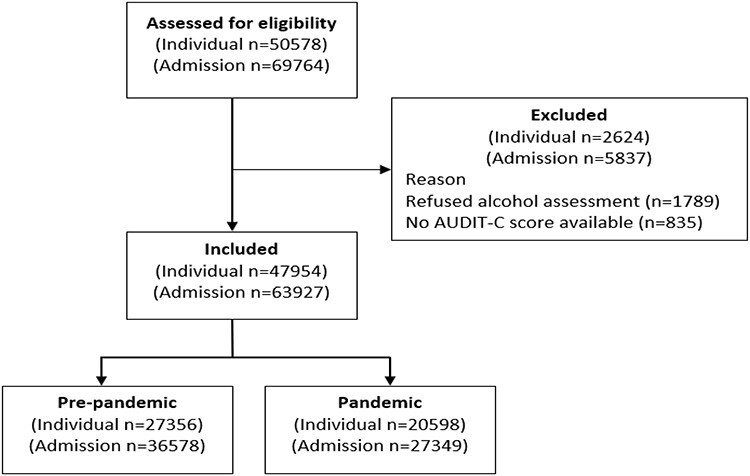
Flow diagram for participant inclusion.

During the study period there were 69,764 admissions to NUH involving 50,578 patients. Of these 1789 (3.5%) declined to complete the alcohol assessment and 835 (1.6%) were excluded for other reasons (Fig. 2). The final study cohort included 63,927 admissions (from 47,954 patients).

The differences between the pre-pandemic and pandemic cohorts are shown in Table 1. There were 36,578 (57.2%) admissions in the pre-pandemic period and significantly fewer, 27,349 (42.8%) in the pandemic period (*P* < 0.001).

**Table 1:** Characteristics of the cohorts

	Pre-pandemic	Pandemic	*P*
All admissions	36,578 (57.2%)	27,349 (42.8%)	<0.001
Individuals	27,356	20,598	
Male	13,024 (47.6%)	10,160 (49.3%)	<0.001
Age years (SD)	63.0 (+/−19.9)	64.0 (+/− 19.7)	<0.001
Ethnicity			0.920
White	19,778 (90.8%)	14,845 (90.7%)	
BAME	2014 (9.2%)	1517 (9.3%)	
Unknown	5564	4236	
IMD quintiles			<0.001
1 (most deprived)	7249 (26.6%)	4571 (23.1%)	
2	4923 (18.0%)	3513 (17.1%)	
3	4635 (17.0%)	3445 (16.8%)	
4	4556 (16.7%)	3639 (17.7%)	
5 (least deprived)	5922 (21.7%)	5200 (25.3%)	
Missing data	71	50	
Civil status			<0.001
In a relationship^a^	13,207 (58.1%)	10,816 (63.3%)	
Not in a relationship^b^	9509 (41.9%)	6276 (36.7%)	
Unknown	4640	3506	
Mode of admission			<0.001
Emergency	15,272 (55.8%)	13,390 (65.0%)	
Other	12,084 (44.2%)	7208 (35.0%)	
Speciality			<0.001
Medicine	13,937 (52.1%)	11,880 (59.4%)	
Surgery	12,791 (47.9%)	8106 (40.6%)	
Other or unknown	628	612	
Length of stay (days)	4 (1–320)	4 (1–173)	<0.001
Number of readmissions	1 (1–17)	1 (1–13)	0.879

Data are *n* (%), mean (SD) or median (range).

^a^In a relationship includes married, in civil partnership or in long term relationship.

^b^Not in a relationship includes single, divorced, separated, dissolved civil partnership, widowed or surviving civil partner.

Those in the pandemic cohort were more likely to be male (49.3% vs 47.6%, *P* < 0.001), older (mean 64.0 vs 63.0 years, *P* < 0.001), more likely to be in a relationship (63.3% vs 58.1%, *P* < 0.001) and were from more affluent socioeconomic quintiles (43.0% vs 38.4%, *P* < 0.001) compared to the pre-pandemic cohort. Ethnic distribution did not differ between the two cohorts.

Patients in the pandemic cohort were more likely to be admitted as an emergency (65.0% vs 55.8%, *P* < 0.001) to a medical speciality (rather than surgical) (59.4% vs 52.1%), *P* < 0.001). Median LOS for the pre-pandemic and pandemic cohorts was unchanged at 4 days. There was no difference in median number of readmissions (median 1, *P* = 0.879) between the cohorts.

### Characteristics of low-risk alcohol drinkers and those with alcohol use disorder

**Fig. 3:**
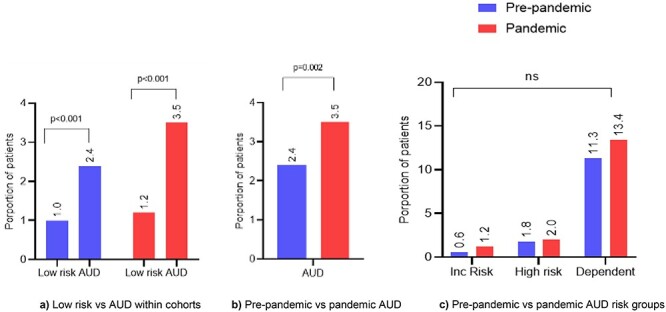
Mental and behavioural disorders due to alcohol.

About 18.4% of the pre-pandemic and 17.6% of the pandemic cohort had AUD. In both cohorts, patients who screened positive for AUD had several shared characteristics. Compared to low risk those who were screened positive for AUD were significantly younger (*P* < 0.001), were more likely to be male (*P* < 0.001), of white ethnicity (*P* < 0.001), have mental and behavioural disorder due to alcohol (*P* < 0.001), less likely to be in a relationship (*P* < 0.001) and cared for by surgical specialities (*P* < 0.001) (Table 2 and Fig. 3).

**Table 2:** Characteristics low risk for AUD and those screening positive for AUD

	Pre-pandemic		*P*	Pandemic		*P*	*P* ^c^
	Low risk	AUD		Low risk	AUD		
All	29,825 (81.6%)	6723 (18.4%)		22,539 (82.4%)	4810 (17.6%)		
Male	9767 (43.5%)	3257 (66.5%)	<0.001	17,098 (49.3%)	2436 (69.6%)	<0.001	0.003
Age years (SD)	64.4 (20.1)	56.3 (17.8)	<0.001	65.1 (19.8)	56.7 (17.7)	<0.001	0.278
Ethnicity			<0.001			<0.001	0.028
White	16,173 (90.0%)	3605 (94.5%)		12,218 (89.7%)	2627 (95.7%)		
BAME	1806 (10.0%)	208 (5.5%)		1400 (10.3%)	117 (4.3%)		
Unknown	4482	1082	3480	756			
IMD quintiles			0.146			0.006	<0.001
1 (most deprived)	5931 (26.5%)	1318 (27.1%)	3995 (23.4%)	756 (21.6%)			
2	4050 (18.1%)	873 (17.9%)		2918 (17.1%)	595 (17.0%)		
3	3864 (17.2%)	771 (15.8%)		2879 (16.9%)	566 (16.2%)		
4	3711 (16.6%)	845 (17.4%)		3027 (17.8%)	612 (17.5%)		
5 (least deprived)	4860 (21.7%)	1062 (21.8%)	4233 (24.8%)	967 (27.7%)			
Missing data	71			50			
Civil status			<0.001			<0.001	<0.001
In a relationship^a^	11,279 (60.3%)	1928 (47.9%)	9283 (65.3%)	1533 (53.2%)			
Not in a relationship^b^	7414 (39.7%)	2095 (52.1%)	4929 (34.7%)	1347 (46.8%)			
Unknown	3768	872		2886	620		
Mode of admission			0.768			0.068	<0.001
Emergency	12,530 (55.8%)	2742 (56.0%)	11,068 (64.7%)	2322 (66.3%)			
Other	9931 (44.2%)	2153 (44%)		6030 (35.3%)	1178 (33.7%)		
Speciality			<0.001			<0.001	<0.001
Medicine	11,719 (53.3%)	2218 (46.6%)	10,065 (60.5%)	1815 (54.3%)			
Surgery	10,252 (46.7%)	2539 (53.4%)	6577 (39.5%)	1529 (45.7%)			
Other or unknown	490	138		456	156		
Length of stay (days)	4 (1–320)	4 (1–178)	<0.001	5 (1–174)	4 (1–135)	<0.001	0.033
Number of readmissions	1 (1–17)	1 (1–11)	<0.001	1 (1–13)	1 (1–13)	0.012	0.0676

Data are *n* (%), mean (SD) or median (range).

^a^In a relationship includes married, in civil partnership or in long term relationship.

^b^Not in a relationship includes single, divorced, separated, dissolved civil partnership, widowed or surviving civil partner.

^c^Significance of difference between pre-pandemic and pandemic AUD.

On comparing AUD screened-positive in the pre-pandemic versus the pandemic cohort, a number of characteristics differed. Those with AUD in the pandemic cohort (as compared to pre-pandemic AUD) were more likely to be of higher socioeconomic background (IMD quintile 1: 21.6% vs 27.1%, IMD quintile 5: 27.7% vs 21.8%, *P* < 0.001), admitted as an emergency (66.3% vs 56.0%, *P* < 0.001), cared for by medical specialities (54.3% vs 46.6%, *P* < 0.001) (Table 2). Patients with AUD in the pandemic cohort had a significantly higher proportion of mental and behavioural disorders due to alcohol (3.5% vs 2.4%, *P* = 0.002) compared to the pre-pandemic cohort (Fig. 3).

On further dividing AUD screened-positive into individual risk groups, significantly higher proportions with AUD in the pandemic cohort were alcohol dependent (3.7% vs 3.0%, *P* < 0.001) compared to pre-pandemic cohort. The variation in proportion for increased risk was 10.4% vs 9.3% (*P* = 0.640), and high risk 5.1% vs 4.7% (*P* = 0.018) for the pre-pandemic and pandemic cohorts, respectively. For both cohorts, the detailed characteristic distribution for each risk group is given in Supplementary Table S1 and distribution for the top 10 inpatients specialities of care in Supplementary Figure SS1.

### Subgroup analysis for pandemic cohort: Covid-19 positive patients

In the pandemic, cohort 994 (4.8%) were diagnosed with Covid-19 infection accounting for 1456 admissions (5.3%). As would be expected, those with Covid-19 infection, overall, were older (mean age 69.0 vs 63.0 years, *P* < 0.001), more likely to be admitted as an emergency (83.1% vs 64.1%, *P* < 0.001), cared for by medical specialities (89.2% vs 57.9%, *P* < 0.001), had longer median length of stay (7 vs 4 days, *P* < 0.001) and more likely to die as an inpatient (26.6% vs 7.6%, *P* < 0.001) compared to those who did not have Covid-19 infection (Supplementary Table SS2).

In those diagnosed with Covid-19 infection, 88 (8.9%) screened positive for AUD. The group with Covid-19 infection and AUD were significantly younger (mean age 62 vs 70 years, *P* < 0.001), more likely to be male (72.7% vs 52.1%, *P* < 0.001), of white ethnicity (98.5% vs 85.0%, *P* < 0.001) and died as an inpatient at a significantly younger age (mean age 63.1 vs 71.6 years, *P* < 0.001) compared to those with Covid-19 infection and screened low risk for AUD. There was no significant difference in index of multiple deprivation IMD, civil status, mode of admission, length of stay, number of readmissions and inpatient mortality between the two groups (Table 3 and Supplementary Fig. S2).

**Table 3:** Characteristics of low-risk vs AUD in Covid-19 positive subgroup in pandemic cohort

	Low risk	AUD	*P*
All	906 (91.1%)	88 (8.9%)	
Male	472 (52.1%)	64 (72.7%)	<0.001
Age (years)	70.0 (18.0)	62.0 (16.0)	<0.001
Ethnicity			<0.001
White	645 (85.0%)	66 (98.5%)	
BAME	114 (15.0%)	1 (1.5%)	
Unknown	147	21	
IMD quintiles			0.941
1 (most deprived)	202 (22.6%)	19 (21.6%)	
2	123 (13.8%)	14 (15.95)	
3	148 (16.6%)	16 (18.2%)	
4	166 (18.65)	17 (19.35)	
5 (least deprived)	254 (28.4%)	22 (25.0%)	
missing	13		
Civil status			0.376
In a relationship^a^	223 (24.6%)	25 (28.4%)	
Not in a relationship^b^	549 (60.6%)	49 (55.7%)	
Unknown	134 (14.8%)	14 (15.9%)	
Mode of admission			0.458
Emergency	750 (82.8%)	76 (86.4%)	
Other	156 (17.2%)	12 (13.65)	
Speciality			0.002
Medicine	804 (88.7%)	76 (86.4%)	
Surgery	106 (10.7%)	1 (1.1%)	
Other or unknown	8 (0.8%)	11 (12.5%)	
Length of stay (days)	7 (1–147)	10 (1–43)	0.112
Number of readmissions	1 (1–8)	1 (1–8)	0.767
Inpatient death	243 (26.8%)	22 (25.0%)	0.801

Data are *n* (%), mean (SD) or median (range).

^a^In a relationship includes married, in civil partnership or in long term relationship.

^b^Not in a relationship includes single, divorced, separated, dissolved civil partnership, widowed or surviving civil partner.

## DISCUSSION

This study demonstrated a largely similar overall pattern of alcohol misuse both pre-pandemic and during the Covid-19 pandemic. However, a significantly higher proportion of hospital admissions during the pandemic were alcohol dependent. Moreover, a significantly higher proportion of patients in the pandemic with AUD had mental and behavioural disorders. Furthermore Covid-19 positive patients with concomitant AUD died as an inpatient at a significant younger age than Covid-19 positive low risk for AUD.

The socioeconomic disparity is a well-described aspect of AUD known as the ‘alcohol harm paradox’. Although people from higher socioeconomic classes drink more alcohol, worse alcohol related outcomes are noted in lower socioeconomic classes—most often amongst young and male populations (Beard *et al*., 2016; Sadler *et al*., 2017). This study demonstrated a similar AUD and mortality trend for age; however, a different socioeconomic disparity distribution was observed between pre-pandemic and pandemic patients screened positive for AUD. One possible explanation for this observation could be easier access to alcohol in more affluent groups whose incomes are not from ‘zero-hours-contracts’. Classically, substance and alcohol misuse has been associated with worse outcomes when a patient has to be admitted to hospital (Roberts *et al*., 2005; Rubin, 2020). Also, the higher proportion of admissions from more affluent areas in the pandemic cohort might be because people in such areas have better access to secondary health care.

AUDIT-C score in a hospital setting identified a higher proportion (18.0%) of inpatients screening positive for AUD than nationally reported figures (7.4%) on alcohol related admissions in 2019 (NHS-Digital, 2020). Others have highlighted that alcohol misuse is disproportionately higher among hospitalized patients but is underdiagnosed and undertreated (Schneekloth *et al*., 2001). Moreover,> there is ongoing concern that a proportion of patients with underlying ARLD are being diagnosed at a very late stage when the scope of any intervention becomes limited (Williams *et al*., 2014). We suggest UAS should become part of routine admission assessment. Patients who screen positive on UAS should be considered for referral to an alcohol care team to minimize future harm. In addition, a linked UAS with routine hospital data can provide a powerful tool to establish targeted alcohol services.

Alcohol misuse is associated with an increased burden of mental health and behavioural disorders. In addition, the Covid-19 pandemic has severely impacted people’s mental wellbeing (Javed *et al*., 2020; Jia *et al*., 2020). There is a mutual relationship between a negative effect on mental health and increasing alcohol intake (Salom *et al*., 2014; The Lancet, 2021). Our findings support commissioning targeted services for mental health and alcohol problems to stem the tide of multi-morbidity highlighted by U.K.’s Royal College of Psychiatry (PSYCH, 2020).

Although half of the pandemic cohort were female, over 70% of those screened positive for AUD were male aged in their 50’s and of white ethnicity which is consistent with reported data defining at risk groups (Booth *et al*., 1992; Gerke *et al*., 1997; Williams *et al*., 2014; Vardy *et al*., 2016; Williams *et al*., 2020). Although 59.4% of the pandemic cohort was admitted under medical specialities, a substantial proportion of increased and high-risk alcohol users (51.0% and 46.0%) were cared for by surgical specialities; predominantly general surgery (13% and 11%) and trauma & orthopaedics (11% and 12.1%). It is notable that the total number of all admissions reduced during the pandemic and was across all risk groups, but a significantly higher proportion presented as an emergency. We suggest that this observed phenomenon among the pandemic cohort could be due to a reduction in routine hospital services as part of pandemic contingency planning.

The key strength of the current paper is the high completion rate of AUDIT-C in the hospital setting (96.5%). This not only significantly reduces the risk of selection bias amongst included participants, but also supports the acceptance of such screening. We acknowledge a few limitations of this study due to its retrospective design. The risk of information and ascertainment bias was mitigated by using an independent person to extract data, unaware of the outcomes. We further assured the validity of the outcome data by risk stratifying alcohol groups using a validated tool (AUDIT-C). The lack of long-term follow up in this study hinders our ability to extrapolate accurate long-term outcome predictions or determine the impact of any natural year-on-year variation. The results may not be generalizable to non-Caucasian populations but likely represent the population trends observed across Europe and high-income countries.

Despite its limitations, the study provides an insight into the relationship between Covid-19, AUD, and demographic characteristics that has implications for policy; in particular the need to identify and manage AUDs in hospital settings. AUDIT-C scoring enhances the correct identification of individuals with harmful alcohol intake who otherwise would have been missed because the primary diagnosis does not always truly represent the extent of alcohol misuse in a hospitalized patient.

## CONCLUSION

Although the pattern of alcohol misuse recorded during the Covid-19 pandemic was largely similar to the pre-pandemic era, a higher proportion of admissions during the pandemic were alcohol dependent. Covid-19 positive patients with concomitant AUD died at a significant younger age than Covid-19 positive at low risk for AUD.

The potential burden of this on healthcare suggests that early intervention opportunities should not be missed. UAS by AUDIT-C score has a high acceptance rate and provides an effective tool to screen hospitalized patients for AUD and identify the change when facing sudden health crises like Covid-19.

## Abbreviations

AUD, alcohol use disorder; ad, alcohol disorders; ARLD, alcohol-related liver disease; CI, confidence interval; CQUIN, Commissioning for Quality and Innovation; SD, standard deviation; ICU, intensive care unit; GCS, Glasgow coma scale; NHS, National Health Services; NICE, The National Institute for Health and Care Excellence; NCEPOD, The National Confidential Enquiry into Patient Outcome and Death; ONS, The Office of National Statistics; PHE, Public Health England; UK, United Kingdom; UAS, Universal alcohol screening

## Supplementary Material

Supplementary_agab059

STROBE_checklist_agab059

## Data Availability

The data underlying this article will be shared at a reasonable request to the corresponding author*.*
