# Supplementary material for: The Effect of Covid-19 on Alcohol Use Disorder and the Role of Universal Alcohol Screening in an Inpatient Setting: A Retrospective Cohort Control Study
Source: Alcohol Alcohol. 2021 Aug 21;57(2):203–10. doi: 10.1093/alcalc/agab059 (PMC8499734; doi:10.1093/alcalc/agab059)
Supplement: Supplementary_agab059 [file supplementary_agab059.docx]

| **SP-Table 1: Pre-pandemic versus Pandemic-cohort comparison between AUD risk groups (Low risk, Increased risk, High Risk and Alcohol dependent)** | | | |
| --- | --- | --- | --- |
|  | **Pre-Pandemic** | **Pandemic** | **p** |
| **Admissions** |  |  |  |
| Low Risk | 29855 (81.6%) | 22539 (82.4%) | 0.001 |
| Increased Risk | 3788 (10.4%) | 2538 (9.3%) | 0.640 |
| High Risk | 1851 (5.1%) | 1273 (4.7%) | 0.018 |
| Dependent | 1084 (3.0%) | 999 (3.7%) | <0.001 |
| **Age** |  |  |  |
| Low Risk | 64.4 (20.1) | 65.1 (19.8) | <0.001 |
| Increased Risk | 57.3 (18.5) | 58.1 (18.7) | 0.190 |
| High Risk | 55.1 (17.5) | 56.4 (17.2) | 0.080 |
| Dependent | 53.9 (14.2) | 52.7 (14.1) | 0.110 |
| **Male** |  |  |  |
| Low Risk | 9767 (43.5%) | 7724 (45.2%) | 0.050 |
| Increased Risk | 1838 (64.0%) | 1304 (67.2%) | 0.020 |
| High Risk | 944 (69.9%) | 706 (73.2%) | 0.720 |
| Dependent | 475 (70.7%) | 426 (71.6%) | 0.070 |
| **Ethnicity white** |  |  |  |
| Low Risk | 16173 (90.0%) | 12218 (89.7%) | 0.490 |
| Increased Risk | 2096 (94.8%) | 1437 (96.1%) | 0.070 |
| High Risk | 1001 (92.9%) | 712 (95.6%) | 0.500 |
| Dependent | 508 (94.5%) | 478 (95.0%) | 0.140 |
| **Civil status in relationship^a^** | |  |  |
| Low Risk | 11279 (60.3%) | 9283 (65.3%) | <0.001 |
| Increased Risk | 1225 (52.8%) | 935 (58.6%) | <0.001 |
| High Risk | 517 (46.2%) | 407 (52.7%) | 0.005 |
| Dependent | 186 (32.0%) | 191 (37.3%) | 0.060 |
| **Emergency mode of admission** | |  |  |
| Low Risk | 12530 (55.8%) | 11068 (64.7%) | <0.001 |
| Increased Risk | 1493 (52.0%) | 1208 (62.3%) | 0.010 |
| High Risk | 763 (56.5%) | 653 (67.7%) | <0.001 |
| Dependent | 486 (72.3%) | 461 (77.5%) | 0.049 |
| **Inpatient speciality medicine** | |  |  |
| Low Risk | 11719 (53.3%) | 10065 (60.5%) | <0.001 |
| Increased Risk | 1176 (42.0%) | 917 (49.4%) | <0.001 |
| High Risk | 613 (46.5%) | 503 (54.3%) | <0.001 |
| Dependent | 429 (66.8%) | 395 (70.7%) | 0.150 |
| **Length of stay** |  |  |  |
| Low Risk | 4 (1-320) | 5 (1-174) | <0.001 |
| Increased Risk | 3 (1-178) | 4 (1-108) | 0..002 |
| High Risk | 4 (1-126) | 4 (1-135) | 0.990 |
| Dependent | 5 (1-131) | 5 (1-68) | 0.150 |
| **Number of readmissions** | |  |  |
| Low Risk | 1 (1-16) | 1 (1-12) | 0.923 |
| Increased Risk | 1 (1-9) | 1 (1-11) | 0.157 |
| High Risk | 1 (1-19) | 1 (1-6) | 0.359 |
| Dependent | 1 (1-10) | 1 (1-12) | 0.299 |

Data is n (%), mean (SD) or median (range)

^a^ In a relationship includes married, in civil partnership or in long term relationship

**SP-Table 2: Characteristics of Covid-19 negative and Covid-19 positive subgroups in pandemic cohort**

|  |  | **Covid-19 negative** | **Covid-19 positive** | ***p*** |  |  |  |
| --- | --- | --- | --- | --- | --- | --- | --- |
| All admissions |  | 25893 (94.7%) | 1456 (5.3%) |  |  |  |  |
| Individuals |  | 19604 (95.2%) | 994 (4.8%) |  |  |  |  |
| Male |  | 9624 (49.1%) | 536 (53.9%) | 0.003 |  |  |  |
| Age years (SD) |  | 63.0 (20.0) | 69.0 (18.0) | <0.001 |  |  |  |
| Ethnicity |  |  |  | <0.001 |  |  |  |
| *White* |  | 14134 (91.0%) | 711 (86.1%) |  |  |  |  |
| *BAME* |  | 1402 (9.0%) | 115 (13.9%) |  |  |  |  |
| *Unknown* |  | 4068 | 168 |  |  |  |  |
| IMD Quantiles |  |  |  | 0.043 |  |  |  |
| *1 (most deprived)* |  | 4530 (23.2%) | 221 (22.5%) |  |  |  |  |
| *2* |  | 3376 (17.3%) | 137 (14.0%) |  |  |  |  |
| *3* |  | 3281 (16.8%) | 164 (16.7%) |  |  |  |  |
| *4* |  | 3456 (17.7%) | 183 (18.7%) |  |  |  |  |
| *5 (least deprived)* |  | 4924 (25.2%) | 276 (28.1%) |  |  |  |  |
| *Missing data* |  | 37 | 13 |  |  |  |  |
| Civil status |  |  |  | <0.001 |  |  |  |
| *In a relationship^a^* |  | 10218 (62.9%) | 598 (70.7%) |  |  |  |  |
| *Not in a relationship^b^* |  | 6028 (37.1%) | 248 (29.3%) |  |  |  |  |
| *Unknown* |  | 3358 | 148 |  |  |  |  |
| Mode of admission |  |  |  | <0.001 |  |  |  |
| *Emergency* |  | 12564 (64.1%) | 826 (83.1%) |  |  |  |  |
| *Other* |  | 7040 (35.9%) | 168 (16.9%) |  |  |  |  |
| Speciality |  |  |  | <0.001 |  |  |  |
| *Medicine* |  | 11000 (57.9%) | 880 (89.2%) |  |  |  |  |
| *Surgery* |  | 8000 (42.1%) | 106 (10.8%) |  |  |  |  |
| Other or unknown |  | 604 | 8 |  |  |  |  |
| Length of Stay (days) |  | 4 (1-174) | 7 (1-147) | <0.001 |  |  |  |
| Number of readmissions |  | 1 (1-13) | 1 (1-8) | <0.001 |  |  |  |
| Inpatient death |  | 1498 (7.6%) | 265 (26.6%) | <0.001 |  |  |  |
| Age at death |  | 76 (13.6) | 78 (11.9) | 0.011 |  |  |  |
| IMD Quantiles for inpatient death | | | | 0.258 |  |  |  |
| *1 (most deprived)* |  | 271 (18.15) | 57 (21.5%) |  |  |  |  |
| *2* |  | 244 (16.35) | 35 (13.2%) |  |  |  |  |
| *3* |  | 262 (17.55) | 46 (17.4%) |  |  |  |  |
| *4* |  | 279 (18.6%) | 56 (21.1%) |  |  |  |  |
| *5 (least deprived)* |  | 439 (29.3%) | 65 (24.5%) |  |  |  |  |
| *Missing data* |  | 3 | 6 |  |  |  |  |

Data is n (%), mean (SD) or median (range)

^a^ In a relationship includes married, in civil partnership or in long term relationship

^b^ Not in a relationship includes single, divorced, separated, dissolved civil partnership, widowed or surviving civil partner

| **SP-Table 3: Inpatient specialities of care** | |
| --- | --- |
| **Medical** | **Surgical** |
| Adult Cystic Fibrosis Service | Blood & Marrow Transplantation |
| Cardiology | Breast Surgery |
| Clinical Haematology | Burns Care |
| Clinical Immunology & Allergy/Dermatology | Cardiac Surgery |
| Clinical Oncology | Cardiothoracic Surgery |
| Diabetic Medicine | Colorectal Surgery |
| Endocrinology | Ear, Nose, Throat (ENT) |
| Gastroenterology | General Surgery |
| General Medicine | Hepatobiliary & Pancreatic Surgery |
| Genitourinary Medicine | Interventional Radiology |
| Health Care for Older People | Maxillo-Facial Surgery |
| Hepatology | Neurosurgery |
| Infectious Diseases | Ophthalmology |
| Medical Oncology | Oral Surgery |
| Nephrology | Plastic Surgery |
| Neurology | Spinal Surgery Service |
| Pain Management | Thoracic Surgery |
| Palliative Medicine | Transplantation Surgery |
| Rehabilitation | Trauma & Orthopaedics |
| Respiratory Medicine | Upper Gastrointestinal |
| Rheumatology | Urology |
| Stroke Medicine | Vascular Surgery |

Obstetrics & gynaecology, intensive care and paediatrics were excluded


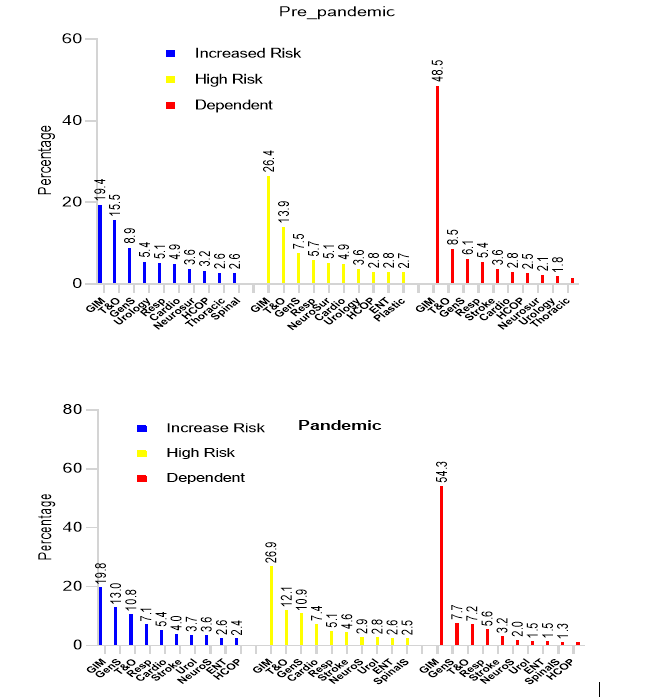
**SP-Figure 1:** Percentage distribution of the top 10 inpatient specialities of care for pre-pandemic versus pandemic cohort (GIM-General Medicine, GenS-General Surgery, T&O-Trauma & orthopaedics, Cardio-Cardiology medicine, Urol-Urology, NeuroS-Neurosurgery, HCOP- Health care of older people, Resp-Respiratory medicine)

**SP-Figure 2: Pandemic-cohort:** Mean age for COVID negative and COVID positive cohort (Neg- all Covid-19 negative group, Pos- all Covid-19 positive group, Died (0-4)- Covid-19 positive patient died inpatient and low-risk for alcohol, Died (5-12)- Covid-19 positive patient died inpatient, and AUDIT-C score ≥5, Alive- Covid-19 positive patient did not die inpatient, Died_IP- Covid-19 positive patient died inpatient) *P ≤0.05 (Significant difference in mean age)
